# The risk of eating disorder relapse during pregnancy and after delivery and postpartum depression among women recovered from eating disorders

**DOI:** 10.1186/s12884-020-03006-7

**Published:** 2020-05-27

**Authors:** Mariko Makino, Mitsuo Yasushi, Sueharu Tsutsui

**Affiliations:** 1 Toho University, School of Psychosomatic medicine, 6-11-1 Omorinishi Ota-ku, Tokyo, Japan; 2 Chuo University, 1-13-27 Kasuga, Bunkyo-ku, Tokyo, Japan

**Keywords:** Eating disorders, Pregnancy, Relapse, Postpartum depression

## Abstract

**Background:**

Studies have shown that women of reproductive age develop eating disorders (EDs). Few studies have examined EDs in women by performing long-term follow-ups during pregnancy and after delivery. Our study aimed to identify relapse of EDs during pregnancy and after delivery as well as postpartum depression in women who had complete remission of EDs.

**Methods:**

Of the 1008 patients with EDs who visited our outpatient clinic between 1994 and 2004, 55 experienced ED remission and pregnancy. Of these, 25 (21 with BN and 4 with AN) consented to participate in this study. Finally, 24 patients were included in this study after 1 patient was excluded owing to a miscarriage. They were interviewed every 2 weeks both during pregnancy and after giving birth. We used the Eating Attitudes Test-26 (EAT-26) and Edinburgh Postnatal Depression Scale (EPDS) as reference scales for diagnosing the EDs and the postpartum depression, respectively. We used a two-sided unpaired test for the statistical analysis.

**Results:**

Sixteen participants (67%) experienced ED relapse during pregnancy and twelve (50%) relapsed after birth. Twelve (50%) had postpartum depression, four of whom (33%) had low-birth-weight infants. Among the participants who did not have postpartum depression, there were no low-body-weight infants. There was no significant difference (*p* = 0.065) in birth weight between the postpartum depression and non-depression groups.

**Conclusions:**

Our study revealed that recurrence of EDs and the occurrence of postpartum depression were higher in this population, indicating the need to closely monitor EDs both during pregnancy and after birth.

## Background

Eating disorders (EDs) are the major mental health disorders that develop during puberty, when adolescents undergo major physical and emotional changes. Claydon et al. [1] reported that pregnancy and the transition to motherhood can be an extremely challenging time for EDs both psychologically and physically. Ward [2] showed that some women develop EDs for the first time during pregnancy, whereas others with a prior history can relapse. Nakai et al. [3] also showed a relationship between EDs and postpartum depression, although more research is required to confirm this relationship. Treatments for pregnant women with EDs and treatments that are effective in preventing relapse in women with EDs require further investigation because such treatments can prevent women with EDs from developing other psychiatric disorders.

In a recent study, Watson et al. [4] showed that having an ED (or history of ED) may result in maternal and infantile complications during pregnancy. Women with a history of EDs may deliver prematurely, their fetuses may not grow properly, and the infants may have low birth weights at delivery.

Only a few long-term follow-up studies have been performed in this field; Suzuki and Takada [5] showed that women who experienced EDs tend to have problems related to marriage, pregnancy, and delivery, long-term follow-ups played an important role in supporting their life.

The strength of our study was that we monitored women with EDs from the beginning of their EDs to delivery continuously with a long follow-up duration.

The present study aimed to identify relapse of EDs during pregnancy and after delivery as well as postpartum depression in women who had complete remission of EDs.

## Methods

The Ethical Committee of the Makino Clinic Ethical approved this study (Ethical Approval Number: 002). The details of the study were described to the patients, and written informed consent was obtained. This study was conducted at the Makino Clinic, 5–1-1- 3F Higashinakano Nakano-ku Tokyo, Japan, which is a private psychiatric clinic closely related to the Toho University hospital, particularly the Department of Psychosomatic Medicine. Patients with psychiatric disorders, such as schizophrenia, depression, panic disorder, adjustment disorder, personality disorder, somatoform disorder, and eating disorder sought consultation at the clinic. Of the 1008 patients with EDs who visited our outpatient clinic between 1994 and 2004, 55 experienced ED remission, pregnancy, and delivery. Of these, 25 (21 with BN and 4 with AN) consented to participate in this study; however, 24 were included in the analysis because 1 patient had a miscarriage. These patients had previously experienced EDs but were in full remission.

We defined remission as 6-month disease-free status. The definition of recovery has already been reported extensively. Levallious et al. [6] defined recovery as a lack of symptoms in the last 90 days. Bardone-Cone et al. [7] defined the absence of symptoms in the past 3 months, whereas Zerwas et al. [8] considered a period of one year. We selected 6 months as a midpoint of the findings in these references.

### Inclusion and exclusion criteria

The Diagnostic and Statistical Manual of Mental Disorders (DSM–5) (American Psychiatric Association, 2013) [9] does not explicitly describe the criteria for ED remission, and in Japan, no specific standardized symptoms are used for the assessment. Therefore, we developed the criteria for the inclusion and exclusion of patients and for defining ED remission based on the literature [10, 11] and after discussion with the patients. We analyzed the patients with full remission for 6 months. The Japanese version of the Eating Attitudes Test (EAT) developed by Ujiie and Kono [12] was used for screening for abnormal eating habits and for diagnosing EDs. Scores of ≥20, 10–19, and ≤ 9 indicated high, low, and no risk of developing EDs, respectively. The reliability and validity of the Japanese version of EAT-26 were also proved by Ujiie and Kono [12]. EAT-26 was also used to investigate the prevalence of EDs in 3023 women in Japan, and it was reported that EAT-26 had high reliability and validity to screen EDs [13].

The Japanese version of the Edinburgh Depression Scale developed by Okano et al. [14] was used to screen for postpartum depression, which was a self-contained scale. Those who scored > 12 were considered to suffer from depression; however, we carefully interviewed those who scored > 9 because at scores over 9 or 10, there were almost no false negatives [14].

### Details of the inclusion and exclusion criteria defining remission from EDs and the interview questions

The interviewer was always the same interviewer. If the patient felt that they were preoccupied with food or wanted to be thinner, etc., the interviewer asked them precisely and structured the interview using the DSM5 criteria of eating disorders for reference.

#### [inclusion criteria → (inclusion and remission criteria)]

EAT ≤9(EAT = Eating Attitudes Test)

Normal eating behavior.

Ability to perform daily activities without restriction.

Some social participation.

No other psychiatric disorders.

Regular menstruation: For patients with AN and regular menstruation, weight gain was not mandatory.

#### [exclusion criteria]

EAT-26 ≥ 9 (EAT = Eating Attitudes Test)

Complications arising from psychiatric disorders such as schizophrenia.

Extreme dependency/obsession of food.

Preoccupied with body shape and weight.

Irregular or no menstruation.

#### [interview questions during pregnancy and after delivery]

Do you feel anxious or depressed about being a mother?

Do you feel you are always preoccupied with foods?

Do you feel really satisfied with your body weight and shape?

Do you have an impulse to eat, binge, and vomit?

Patients were interviewed every 2 weeks both during pregnancy and after giving birth. After these “Yes/No” questions, patients were asked if they have any physical, psychological, or social concerns or problems. In the beginning, “Yes/No” questions were effective because they felt nervous to some extent.

### Relapse group

Nausea, binge, and vomit were similar to those observed in hyperemesis or pregnancy sickness; however, if the patients felt that they wanted to lose weight and be thinner, these feelings were very different from those in hyperemesis. Thus, we termed this group relapse group. If their symptoms stopped during pregnancy, we termed this temporary relapse group.

### Statistical analysis

Statistical analysis was performed using a two-sided unpaired test. In statistics, Welch’s t-test or unequal variances test is a two-sample location test which is used to test the hypothesis that two populations have equal means. Welch’s t-test is an adaptation of Student’s t-test and is more reliable when the two samples have equal variances and/or unequal sample sizes.

We compared the findings between the temporary ED relapsing group and the non-relapsing group during pregnancy and after delivery. We also compared the findings between the postpartum depression and the non-depression group within 3 months of delivery. A *P* value of < 0.05 was considered statistically significant, whereas a *P* value of < 0.1 was considered marginally significant.

## Results

### Information of the patients

The characteristics of the women who recovered from EDs and who were interviewed during pregnancy and after birth are presented in Table 1.

**Table 1:** Characteristics of women* who recovered from EDs and were interviewed during pregnancy and after birth

Age at onset of diagnosis (years)	16.6 (SD 3.3)
Disease duration (years)	9.5 (SD 5.4)
Age at remission (years)	26.1 (SD 5.3)
Maternal age (years)	28.1 (SD 5.4)
Temporary ED relapse during pregnancy (%)	16 (67%)
Gestational age at delivery (week)	39 (SD 1.1)
Complications (%)	17 (67%)
Complications in the infants (%)	3 (13%)
Vaginal delivery (%)	19 (79%)
Infant weight (g)	2928 (SD 540)
ED relapse after delivery (%)	12 (50%)
Postpartum depression (%)	12 (50%)
ED relapse after delivery (%)	12 (50%)
Postpartum depression (%)	12 (50%)

* ***n*** **= 24:** 1 patient had miscarriage; hence, we analyzed 24 patients

Age: maternal age

### Temporary ED relapse during pregnancy

Of the 24 patients in this study, 16 patients showed temporary relapse within 3 months after conceiving. None of them maintained their EDs throughout the pregnancy. We named this relapsing group with the 16 patients as the temporary relapsing group and the remaining 8 patients were classified as the non-relapsing group.

Table 2 shows a comparison of the findings between the temporary ED relapsing group and the non-relapsing group during pregnancy.

**Table 2:** Comparison between the temporary ED relapsing group (detected during pregnancy) and non-relapsing group during pregnancy

	Temporary ED relapsing group (*n* = 16)	Non-relapsing group (*n* = 8)	
Age at onset of diagnosis (years)	15.9 (SD 1.2)	18.0 (SD 5.4)	NS
Disease duration (years)	9.1 (SD 4.0)	10.3 (SD 7.9)	NS
Age at remission (years)	25.1 (SD 4.4)	28.3 (SD 6.5)	NS
Age at pregnancy (years)	27.1 (SD 5.0)	30.1 (SD 5.8)	NS
Gestational age at delivery (weeks)	38.3 (SD 1.3)	39 (SD 0.5)	NS
Maternal complications (%)	12 (75%)	6 (75%)	NS
Problems in the infant (%)	6 (12.5%)	1 (12.5%)	NS
Vaginal delivery (%)	11 (68.8%%)	8 (100.0%%)	NS
Infant weight (g)	2763 (SD 392)	3259 (SD 664)	*P* < 0.1
Postpartum depression (%)	10 (62.5%)	2 (25.0%)	NS
ED relapse after delivery (%)	11 (68.8%)	1 (12.5%)	NS
Postpartum depression (%)	10 (62.5%)	2 (25.0%)	
ED relapse after delivery (%)	11 (68.8%)	1 (12.5%)	*P* < 0.05

### Postpartum depression and comparison between the temporary ED relapsing and non-relapsing groups after delivery

The rate of the postpartum depression was the same for these two groups.

Table 3 shows the differences between the ED relapsing group and the non-relapsing group after delivery.

**Table 3:** Comparison between the ED relapsing group (detected during pregnancy) and non-relapsing group after delivery

	Temporary ED relapsing group *n* = 12	Non-relapsing group n = 12	
Age at onset of diagnosis (years)	15.9 (SD 1.4)	17.3 (SD 4.4)	NS
Disease duration (years)	7.5 (SD 3.0)	11.5 (SD 6.6)	NS
Age at remission (years)	23.4 (SD 3.9)	28.8 (SD 5.2)	P < 0.05
Age at pregnancy (years)	24.8(SD 4.1)	31.5 (SD 4.3)	P < 0.05
Gestational age at delivery (weeks)	38.3 (SD 1.4)	38.8 (SD 0.7)	NS
ED relapse during pregnancy (%)	11 (91.7%)	5 (41.7%)	P < 0.05
Maternal Complications	9 (75%)	9 (75%)	NS
Problems in the infant	2 (12.5%)	1 (12.5%)	NS
Vaginal delivery	7 (58.3%)	12 (100%)	P < 0.1
Infant weight (g)	2813 (446)	3044 (618)	NS
Postpartum depression	6 (50%)	6 (50%)	NS

### Complications in the pregnancy

Our results showed various complications such as 5 patients with diabetes mellitus, 3 with anemia, 2 threatened with miscarriage, 2 with kidney stones, 1 with nephrosis, 1 with eclampsia, 1 with hypertension, 1 with placenta previa, and 1 with miscarriage, and 5 underwent cesarean sections. In spite of the small sample size, there were many complications.

### Comparison of the postpartum depression and non-depression groups

Table 4 shows the comparison between the postpartum depression and non-depression groups. The only difference was in the infant body weight.

**Table 4:** Comparison between postpartum depression group and non-depression group

	Depression group (n = 12)	Non-depression group (n = 12)	Level of significance
Age at onset of diagnosis (years)	15.8 (SD 0.8)	17.4 (SD 4.5)	NS
Disease duration (years)	10.4 (SD 3.3)	8.6 (SD 7.6)	NS
Age at remission (years)	26.3 (SD 3.4)	26.0 (SD 6.9)	NS
Age at pregnancy (years)	28.3 (SD 3.4)	27.9 (SD 6.8)	NS
Gestational age at delivery (week)	38.4 (SD 1.3)	38.8 (SD0.9)	NS
Temporary ED relapse during pregnancy (%)	10 (83.3%)	6 (50%)	NS
Maternal complications (%)	7 (58.3%)	9 (75%)	NS
Problems in the infant (%)	2 (16.7%)	1 (8.3%)	NS
Vaginal delivery (%)	11 (91.7%)	8 (66.7%)	NS
Infant weight (g)	2669 (SD 406)	3188 (SD 544)	P < 0.1
ED relapse after delivery (%)	6 (50%)	6 (50%)	NS

## Discussion

We predicted that EDs were influenced by pregnancy and delivery such as suffering from ED relapse during pregnancy and after delivery and postpartum depression, and our hypothesis was confirmed by the findings of our study.

We found that 67% of these patients experienced a temporary relapse of the EDs within the first 3 months after conceiving but they recovered after counseling. We found that irrespective of the relapse, the maternal and infant complications were the same. However, the incidence of postpartum depression was lesser and the infant weight was higher in the non-relapsing group. After the delivery, the rate of postpartum depression and complications were found to be the same in the relapsing and the non-relapsing group. Further, the infants in the postpartum depression group had lesser body weights than those of the non-depression group.

Angela [15] reported that ED relapse was common and Hetman et al. [16] showed that even professionals could not always predict who was vulnerable to relapse if patients with EDs had an opportunity to stabilize weight on the completion of treatment, if the completion of treatment would trigger relapse, leaving the patient vulnerable to an unhealthy weight gain/loss, and if relapse could occur during transition phases such as starting school or college, starting a new job, becoming pregnant, having a baby, or becoming a parent. Thus, pregnant patients with EDs should undergo follow-up during pregnancy and the postpartum period.

We followed up our patients from the onset of the disease to the pregnancy and postpartum period. They regularly visited our clinic and we encouraged them how to control their eating behavior and emotions. There are some prevention approaches for EDs. Kelty Mental Health Center in British Columbia Provincial Health Service Authority [17] showed that relapse could be prevented by developing a support system including family members, reducing negative influences, identifying triggers, creating a personal coping plan, and eating snacks and meals regularly. Our approach enabled them to change and have good self-control and relationships with the others, including their partners and families. The targets were similar to those of Kelty. We found that regular follow-ups of patients with EDs are one of the prevention strategies for EDs, including pregnant women with EDs.

Morgan and Russel [18] reported that EDs during pregnancy indicated ED relapse. Our study indicated all patients with AN and 48% of the patients with BN had temporary ED relapse during pregnancy, which is consistent with that reported in previous studies [2, 5]. Morgan et al. [18] also showed that compared to patients with BN, those with AN were more conscious of their body shape. Volpe et al. [19] reported that patients with AN have compulsive tendencies, which can trigger relapse. Although our sample size was very small, all patients with AN had temporary relapse during their pregnancy, which may explain their compulsive tendencies. Pregnancy for many women can be a happy and relaxing time in their life. However, even they are happy, they may be afraid of gaining weight and facing body shape changes. Moreover, people with a history of EDs could be worried about gaining weight and it may trigger the ED symptoms. Hetman et al. [16] reported that pregnancy is one of the risk factors for the relapse of the EDs. Patients with a history of AN tend to be at a high risk for relapse, because AN relapse stems from a desire to lose weight and maintain body shape (by reducing food intake) during the pregnancy, despite the desire to be pregnant. Similarly, Mancini [20] reported that body image dissatisfaction is common in pregnant women. Our results suggest that patients with a history of EDs and body image dissatisfaction had a greater risk of relapse.

The patients in our study experienced various complications during the pregnancy. Ekeus et al. [21] reported that women with BN have a higher risk of miscarriage. Our results showed that one woman with a history of BN had a miscarriage. Koubaa et al. [22] reported that in patients with AN, fetuses experience poor intrauterine growth, and women deliver low-birth-weight infants. The Japanese Nikkei Health [23] reported that an average infant’s body weight should be 2980 g for males and 2910 g for females. Our results showed that the average infant body weight was 2928 g ± 540 g. There was no significant difference in the results in our study compared with those of the Nikkei Health report in Japan. At delivery, in Japanese, the average infant body weight was 3040 g in male infants and 2960 g in female infants [23]. We reported that infant body weight was lesser in the temporary relapsing group than in the non-relapsing group because the temporary relapsing group reported that they consumed less food and vomited after eating, possibly leading to insufficient weight gain. However temporary relapse group showed recovery within the first 3 months of pregnancy. The reason behind this was unclear. Infant body weight of the temporary relapsing group was lesser than that of the non-relapsing group. However, infant body weight of this study showed no significant difference compared with the average infant weight in Japan. It may be said that the first 3 months of the pregnancy are not crucial for the final weight of the baby. Watson et al. [4] reported about complications arising in infants for a very large sample study. They showed that infant complications included low birth weight and height causing a risk for growth of the infants. They showed that EDs had high risk for negative health outcomes in pregnant ED women and their babies.

According to the Barker hypothesis, intrauterine growth restriction or low birth weight has a causal relationship with the origin of hypertension, coronary heart disease, and non–insulin-dependent diabetes in middle age [24]. Thus, follow-up of low-body-weight infants may be important to verify the Barker hypothesis.

Franco et al. [25] found that the majority of the women with active EDs had normal pregnancies, resulting in healthy babies. Our results might be similar to those of Franco et al. It may be said that infants of women with temporary ED relapse during pregnancy or of those with a history of EDs tend to have normal weight. Further, our patients did not have active EDs; therefore, when they gave birth, their babies could be healthier than those from mothers with active EDs. Angela et al. [26] reported that there were specific complications in mothers with EDs, including maternal anemia, diabetes mellitus (DM), and placental infarction. Our findings corroborated this report. This time infants were observed by pediatricians. We did not exact result of what would become of the infants.

Bennet et al. [27] **s**howed that 50% of the patients with EDs had postpartum depression and that the prevalence in the general population was approximately 13%. Our results agree with those of that study because 50% of patients with postpartum depression had a history of EDs. Nasreen et al. [28] reported that maternal depression was associated with low birth weight in infants. Our result also showed that after delivery, infants of women in the depression group had less body weight than those in the non-depression group.

Owing to the small sample size, we cannot conclude that patients with BN had a higher risk to develop postpartum depression. However, since approximately 50% of the patients with BN had postpartum depression, BN may lead to a higher risk of postpartum depression. With regard to AN, 3 out of the 4 patients had postpartum depression, but the reason is unclear. Volpe et al. [29] reported that patients with BN can develop depression within 12 months post-delivery; some patients with EDs achieve remission and emotional stability while others show relapse. Mazzeo et al. [30] showed that those with a history of EDs have a greater risk of relapse during pregnancy, thereby leading to postpartum depression and heightened anxiety. Chan et al. [31] reported that higher levels of disordered eating in pregnancy were significantly associated with higher levels of postpartum depression. However, our data did not show that the non-relapsing group had lesser postpartum depression than the temporary ED relapsing group. Overall, compared with healthy women, pregnant women with a history of ED had a higher prevalence of postpartum depression and they required long-term follow-up care. In our data, 50% of the patients experienced ED relapse within one year of delivery. Morgan et al. [18] reported that 66% of patients with ED history had bulimic symptoms after delivery. Unfortunately, we do not have the precise data on the relapse types of EDs after delivery, and therefore, we could not confirm the findings of Morgan et al. Patients attended our outpatient clinic every 2 weeks and received counseling regarding how to manage their stress and anxiety levels, which may have helped in preventing the relapse of the EDs.

There were a number of limitations in this study. Our study had a small sample size. A larger group size would have increased the power and allowed for comparisons between the groups with EDs. Although our results could have supported our hypothesis that women with a history of ED are susceptible to relapse during pregnancy and after delivery and having postpartum depression, our samples were limited to only two groups: women with AN and women with BN. Other subtypes of EDs should also be analyzed to generalize our results. The most important limitation in this study was the absence of a control group that had no EDs (healthy women). The information and the results of our study were extracted rather than planned for the longitudinal study.

In the future, before researching the outcomes of the women with EDs related to pregnancy and after delivery, we have to prepare larger sample sizes of both women with past EDs and healthy women. Further, we have to approach the department of Obstetrics and Gynecology and Pediatrics and monitor the infants’ growth, relationship between mothers and infants, including breastfeeding problems, and the role of family relationships. For predicting postpartum depression, we have to address specific questions, including the social support and the feeling of being a mother and how they feel about their body shape, because Chan et al. [31] suggested that body dissatisfaction was significantly associated with postpartum anxiety and depressive symptoms for 6 months after the delivery. In future, we should carefully investigate the feelings of these mothers regarding their body dissatisfaction and body image after delivery and analyze the association between postpartum depression and body image.

## Conclusions

We arrived at several conclusions on the basis of follow-up for EDs. Our study revealed that the recurrence of EDs and occurrence of postpartum depression were higher in our study population (24 women with EDs who had recovered), indicating that EDs need to be closely monitored during pregnancy and after giving birth.

## Supplementary information

**Additional file 1.** Patients’ Characteristics. Tabular description of the demographic data of the participants.

## Data Availability

All data generated or analyzed during this study are included in this published article.

## Abbreviations

AN: Anorexia nervosa
BN: Bulimia nervosa
DM: Diabetes mellitus
EAT: Eating Attitudes Test
ED: Eating disorders
EPDS: Edinburgh Postnatal Depression Scale
