# Supplementary material for: The risk of eating disorder relapse during pregnancy and after delivery and postpartum depression among women recovered from eating disorders
Source: BMC Pregnancy Childbirth. 2020 May 27;20:323. doi: 10.1186/s12884-020-03006-7 (PMC7251919; doi:10.1186/s12884-020-03006-7)
Supplement: Supplementary file 1 — Additional file 1. Patients’ Characteristics. Tabular description of the demographic data of the participants. [file 12884_2020_3006_MOESM1_ESM.docx]

Additional file 1. Patients’ Characteristics

| Participants | Age at onset | Age at | Age at pregnancy | Weeks of | Complications | Problems in the infant | Advocate | Method of delivery | Weight & gender of the infant | Temporary ed relapse during pregnancy | Postpartum depression | Postpartum relapse of ed |
| --- | --- | --- | --- | --- | --- | --- | --- | --- | --- | --- | --- | --- |
|  | diagnosis | remission |  | gestation |  |  |  |  |  |  |  |  |
| 1 | 16 BNP | 30 | 34 | 38 | threatened miscarriage | No | No | vaginal delivery | 2700 | binge | yes | No |
|  |  |  |  |  |  |  |  |  | Male | Vomit |  |  |
| 2 | 15 BNP | 25 | 27 | 40 | DM | No | Husband biological mother | vaginal delivery | 3333 | Binge | Yes | Yes |
|  |  |  |  |  |  |  |  |  | Male | Vomit |  |  |
| 3 | 16 BNP | 27 | 29 | 39 | Nephrosis | No | Biological Mother | Vaginal delivery | 2366 | No | Yes | No |
|  |  |  |  |  |  |  |  |  | Male |  |  |  |
| 4 | 16　BNP | 24 | 27 | 39 | Kidney stone | No | Husband | Vaginal delivery | 2902 | No | Yes | No |
|  |  |  |  |  |  |  |  |  | Male |  |  |  |
| 5 | 18 BNP | 25 | 28 | 39 | Eclampsia | No | Biological | Vaginal delivery | 3466 | No | No | No |
|  |  |  |  |  |  |  | Mother |  | Male |  |  |  |
|  |  |  |  |  |  |  | Husband |  |  |  |  |  |
| 6 | 16 BNP | 20 | 23 | 40 | No | No | Biological mother | Vaginal delivery | 2568 | Binge | Yes | Yes |
|  |  |  |  |  |  |  | Husband |  | Male |  |  |  |
| 7 | 15 BNP | 30 | 33 | 39 | No | No | Biological mother | Vaginal delivery | 2400 | AN | Yes | No |
|  |  |  |  |  |  |  |  |  | Male |  |  |  |
| 8 | 16 BNP | 30 | 32 | 38 | No | No | Biological | Vaginal delivery | 2700 | AN | Yes | No |
|  |  |  |  |  |  |  | mother |  | Male |  |  |  |
| 9 | 16 BNP | 30 | 32 | 38 | Threatened miscarriage | No | No | Vaginal delivery | 2710 | Binge | Yes | No |
|  |  |  |  |  |  |  |  |  | Male | Vomit |  |  |
| 10 | 15 BNP | 25 | 27 | 36 | No | Low birth weight | Husband | Vaginal | 2200 | Binge | Yes | Yes |
|  |  |  |  |  |  |  | Biological mother | delivery | Female | Vomit |  |  |
| 11 | 15 BNP | 22 | 23 | 36 | DM | Low birth weight | Biological mother | Vaginal delivery | 2100 | Binge | Yes | Yes |
|  |  |  |  |  |  |  |  |  | Female | Vomit |  |  |
| 12 | 14 BNNP | 28 | 32 | 39 | Kidney stone | No | Biological | Vaginal delivery | 3450 | No | No | No |
|  |  |  |  |  |  |  | mother |  | Male |  |  |  |
| 13 | 18 BNNP | 25 | 27 | 39 | Hypertension | No | Husband | Vaginal delivery | 3520 | No | No | No |
|  |  |  |  |  |  |  | Biological mother |  | Male |  |  |  |
| 14 | 16 BNP | 43 | 43 | 40 | DM | Overweight | Husband | Vaginal delivery | 4500 | No | No | No |
|  |  |  |  |  |  |  |  |  | Male |  |  |  |
| 15 | 31 BNP | 31 | 31 | 38 | No | No | Biological mother | Vaginal delivery | 2568 | No | No | No |
|  |  |  |  |  |  |  |  |  | Male |  |  |  |
| 16 | 15 BNNP | 17 | 18 | 37 | Anemia | No | Biological | Caesarean | 2460 | AN | No | Yes |
|  |  |  |  |  |  |  | mother | section | Female |  |  |  |
| 17 | 15 BNNP | 23 | 24 | 39 | DM | No | Husband | Vaginal delivery | 3300 | No | No | Yes |
|  |  |  |  |  |  |  |  |  | Male |  |  |  |
| 18 | 16 BNNP | 25 | 25 | 39 | No | No | Husband | Vaginal delivery | 3450 | Binge | Yes | Yes |
|  |  |  |  |  |  |  |  |  | Male | Vomit |  |  |
| 19 | 16 BNP | 24 | 24 | 38 | DM | No | Biological | Caesarian | 2903 | Binge | No | Yes |
|  |  |  |  |  |  |  | mother | section | Female | Alcohol |  |  |
| 20 | 16 BNP | 23 | 30 | 40 | No | No | Biological mother | Vaginal delivery | 3250 | Binge | No | No |
|  |  |  |  |  |  |  |  |  | Female |  |  |  |
| 21 | 18 BNP | 24 | 25 | 20 | Miscarriage |  |  |  |  |  |  |  |
| 22 | 18 ANP | 30 | 32 | 38 | Placenta previa | No | No | Caesarean section | 2785 | AN | No | Yes |
|  |  |  |  |  |  |  |  |  | Male |  |  |  |
| 23 | 18 ANNP | 26 | 28 | 39 | Anemia | No | Biological mother | Vaginal delivery | 2856 | Binge | No | Yes |
|  |  |  |  |  |  |  |  |  | Female | Vomit |  |  |
| 24 | 18 ANNP | 27 | 28 | 39 | Anemia | No | Biological mother | Caesarean section | 2600 | AN | Yes | Yes |
|  |  |  |  |  |  |  |  |  | Female |  |  |  |
| 25 | 14 ANNP | 17 | 18 | 38 | No | No | Biological mother | Caesarean section | 3200 | Binge | No | Yes |
|  |  |  |  |  |  |  |  |  | Female | Vomit |  |  |

Note:

AN=anorexia nervosa,

BN= Bulimia Nervosa,

P=purging,

NP=not purging,

DM= diabetes mellitus
